# Symptoms, antibody levels and vaccination attitude after asymptomatic to moderate COVID-19 infection in 200 healthcare workers

**DOI:** 10.3205/dgkh000386

**Published:** 2021-04-19

**Authors:** Igor Alexander Harsch, Andrea Ortloff, Mike Reinhöfer, Jörg Epstude

**Affiliations:** 1 Thuringia Clinic Saalfeld “Georgius Agricola”, Saalfeld/Saale, Germany; 2 MVZ DIANOVIS, Greiz, Germany

**Keywords:** COVID-19, COVID-19 antibodies, vaccination, vaccination decision, cutaneous hyperesthesia

## Abstract

**Aim:** In Germany, the willingness to be vaccinated against COVID-19 is rather low among medical staff. We collected data on symptoms, antibody titers and vaccination readiness from clinic employees at a municipal clinic who had already been through a COVID-19 infection (asymptomatic to moderate). We also examined the antibody titers for their possible importance as an individual decision-making aid with regard to vaccination.

**Method:** 200 employees of our municipal clinics were included in the study. COVID-19 antibody determination was performed using an ELISA (EUROIMMUN™, PerkinElmer, Inc. Company). The participants were given an anonymous questionnaire containing anthropometrical issues, symptoms of the infection and questions concerning the vaccination decision. Finally, the antibody levels were reported to the participants and the attitude towards a vaccination was reevaluated.

**Results:** In all 200 participants who had already gone through a COVID-19 infection, 75 employees were in favor of a vaccination (37.5%), 96 were opposed to vaccination (48%), and 29 were undecided (14.5%). In the different occupational groups, the positive trend in terms of willingness to be vaccinated was highest among physicians and is least among nurses. The antibody results showed considerable variation in titer levels and therefore did not correlate with disease severity in asymptomatic to moderately ill persons. We also observed a pro-vaccination trend with increasing age of the participants. The specifically-asked symptom of cutaneous hyperesthesia during COVID-19 infection occurred in 5% of the participants.

**Conclusion:** In medical personnel who had already suffered from a COVID-19 infection, the willingness to receive a vaccination tends to be highest among physicians, and lowest in nurses, and increases with age. For the vast majority of those affected, knowledge of the antibody titers only reinforces the vaccination decision made beforehand and thus does not contribute to a change in vaccination decision. The specifically-requested symptom of cutaneous hyperesthesia during COVID-19 infection was unexpectedly frequent.

## Zusammenfassung

**Zielsetzung:** In Deutschland ist die Impfbereitschaft des medizinischen Personals vergleichsweise gering. Wir haben Daten zu Symptomen, Antikörpertitern und zur Impfbereitschaft von Mitarbeitern in einer städtischen Klinik gesammelt, die bereits eine COVID-19-Infektion (asymptomatisch bis mittelschwer) durchgemacht hatten. Wir haben auch die Bedeutung der Antikörpertiter auf ihre mögliche Rolle als individuelle Entscheidungshilfe im Hinblick auf die Impfung untersucht.

**Methoden:** 200 Mitarbeiter unserer städtischen Kliniken wurden in die Studie einbezogen. Die Antikörperbestimmung gegen COVID-19 wurde unter Verwendung eines ELISA (EUROIMMUN™, PerkinElmer, Inc. Company) durchgeführt. Den Teilnehmern wurde ein anonymer Fragebogen ausgehändigt, der anthropometrische Fragen, Symptome der Infektion und Fragen zur Impfentscheidung enthielt. Schließlich wurden die Antikörperwerte mit den Teilnehmern diskutiert und die Einstellung zu einer Impfung erfragt.

**Ergebnisse:** Von den 200 Teilnehmern, die bereits eine COVID-19-Infektion hatten, befürworteten 75 Mitarbeiter eine Impfung (37,5%), 96 waren gegen eine Impfung (48%) und 29 konnten keine endgültige Entscheidung treffen (14,5%). In den verschiedenen Berufsgruppen ist der positive Trend in Bezug auf die Impfbereitschaft besonders bei Ärzten ausgeprägt, und die Impfbereitschaft scheint in der Pflege am niedrigsten zu sein. Die Antikörperbestimmungen zeigten eine beträchtliche Variabilität der Titerwerte und korrelieren nicht mit der Krankheitsintensität in einer asymptomatischen bis mittelschwer erkrankten Personengruppe. Wir beobachteten einen positiven Trend zur Impfung mit zunehmendem Alter der Teilnehmer. Das explizit erfragte Symptom einer cutanen Hyperästhesie während einer COVID-19-Infektion trat bei 5% der Teilnehmer auf.

**Schlussfolgerung:** Bei medizinischem Personal, das bereits an einer COVID-19-Infektion gelitten hat, ist die Bereitschaft, eine Impfung zu erhalten, bei Ärzten am höchsten, in der Pflege am niedrigsten und steigt mit zunehmendem Alter. Für die überwiegende Mehrheit der Betroffenen zementiert die Kenntnis der Antikörpertiter nur die zuvor getroffene Impfentscheidung und trägt somit nicht zu einer Änderung der Impfentscheidung bei. Das speziell erfragte Symptom einer Hauthyperästhesie während einer COVID-19-Infektion ist unerwartet häufig.

## Introduction

The various problems raised by vaccination against COVID-19 include three points, some of which are addressed in the following investigation:

1. There is currently a shortage of vaccines.
2. The willingness to be vaccinated among the medical and nursing staff prioritized in Germany leaves much room for improvement [1], [2], [3].
  To date, there are only a few peer-reviewed studies on the willingness to be vaccinated against COVID-19 in the medical and nursing fields in the German-speaking area. The German Society for Internal Intensive Care Medicine and Emergency Medicine and the German Interdisciplinary Association for Intensive Care and Emergency Medicine conducted an anonymous online survey in December 2020 of medical staff, mainly intensive care staff, about their willingness to be vaccinated. A total of 2,305 people took part in the survey. The nursing staff of intensive care units were significantly less willing to be vaccinated than were the physicians [1], [2]. The unwillingness to be vaccinated generally seems to be more pronounced among nursing staff than among physicians [3].
3. The question of whether a vaccination also makes sense for people who have already had COVID-19 infection could not be answered in January 2021 by the Center for Disease Control, RKI and STIKO (The Robert Koch Institute [RKI] is the German federal government’s central institution in the field of disease monitoring and prevention. The STIKO [Ständige Impfkommission am Robert Koch-Institut] [Standing Committee on Vaccination] is an expert group located at the RKI which deals with health policy questions about vaccinations and infectious diseases, and issues corresponding recommendations) [4].
  In addition, there are reports about reinfections after a COVID-19 infection [5], which are unsettling especially for laypeople.

The data situation regarding immunity after a past COVID-19 infection is controversial. After the REACT study from England indicated a rapid decline in seroprevalence of antibodies [6], a more optimistic assessment was recently published [7]. The majority of the examined patients had a “robust” immune response with neutralizing antibodies, which was still detectable even after 5 months.

As mentioned above, it had not been clarified as of January 2^nd^, 2021 whether a person who had already suffered COVID-19 and recovered should be advised to get vaccinated with a COVID-19 vaccine when available. The statement of the CDC (accessed January 2^nd^, 2021) was: “There is not enough information currently available to say if or for how long after infection someone is protected from getting COVID-19 again; this is called natural immunity […]. CDC cannot comment on whether people who had COVID-19 should get a COVID-19 vaccine“ [4].

The Statement of RKI and STIKO was comparable until January 14^th^ (when the study design was already finished) and was then adapted to: “The STIKO therefore sees the need for a booster vaccination even after a SARS-CoV-2 infection. However, the appropriate time for this cannot yet be specified”. In the update from January 29^th^, 2021, a vaccination six months after past COVID-19 infection is recommended [8].

Given this background, we were interested in the willingness to be vaccinated among employees of our clinic who had already been infected with COVID-19, in terms of the different occupational groups and age. Since these employees had asymptomatic to moderate courses of the infection, we were also interested in their symptoms, antibody levels, and the impact of the antibody titers on the decision for or against vaccination, as well as their sources of information.

## Materials and methods

After obtaining informed consent and with approval by the Ethics Committee of the State Medical Association of Thuringia, we examined 205 employees of the Thuringia Clinics working at the three locations (Saalfeld, Rudolstadt, Pößneck) who had already gone through a COVID-19 infection in the second wave of the pandemic (i.e., in our clinic beginning in September 8^th^, 2020) 3–4 weeks or more prior to the study. The infection had to be confirmed by PCR, and the course had to be asymptomatic, mild or moderate according to the NIH criteria [9].

In addition to the declaration of consent, the participants were given an anonymous questionnaire. It contained questions on age, gender, occupation, time since recovery from the infection, questions about a presumed source of infection (several answers possible), as well as the questions: “Would you make your decision for or against a vaccination dependent on your antibody levels?” and “Have you already made your decision, but are primarily interested in the antibody levels?”. In addition, the symptoms of the past infection were queried in order to assess the severity of the past infection according to the NIH classification. These items were supplemented by a question on occurrence, location and duration of cutaneous hyperesthesia.

Blood was then taken to determine the IgA and IgG titers against COVID-19. For antibody determination against SARS-CoV-2, we used an ELISA (EUROIMMUN™, a PerkinElmer, Inc. company), which is CE (Conformité Européenne)-certified and IVD (In Vitro Diagnostic)-approved, and additionally validated in-house by the DIANOVIS lab. The specifity of the test for IgG is specified at 98.5% by the distributor and at 92.5% for IgA. Antibody titers below 0.8 are scored “negative”, and after discussion with the laboratory doctors, we considered titers of ≥2 to be reliable and significant.

The antibody levels were reported to the participants usually 2–4 weeks after filling out the questionnaires. They were also informed that the humoral immune system is responsible for formation of antibodies, but that another important branch of the immune system is the cellular immune response. They were also informed that the latter part of the immune system is also stimulated by a vaccination, and that it is thus possible that a protective immune response can be achieved by vaccination even in the case of insufficient antibody generation (IAH, end of study March 12^th^, 2021). 

The state of knowledge, which is still very much in flux, was also addressed. For example, the STIKO statement in early January 2021 that, according to experts, “[…] a vaccination may be not absolutely necessary after an infection has passed […]” was changed to “The STIKO therefore sees the need for a booster vaccination even after a SARS-CoV-2 infection.” The test persons were informed that reassessment and revision of this statement are possible, according to the changing state of knowledge. The latest statement – that this “booster” should take place 6 months after the infection – has been completed was also published on the clinic’s internal website.

In view of the antibody titers that were now available, the test subjects were asked whether they were ready to be vaccinated and what influenced their decision. 

## Results

### Characteristics of the study participants

200 employees met the inclusion criteria, 5 participants from the initial group were excluded, since they had experienced severe illness or for other reasons. Characteristics of the participants are given in Table 1 (Tab. 1).

### Symptom of cutaneous hyperesthesia

In view of the previous individual case descriptions of cutaneous hyperesthesia in COVID-19 infection, we took the opportunity to specifically ask about this symptom in a larger cohort. These symptoms were reported in 10 people, which allows the cautious conclusion of an incidence of 5% in clinically asymptomatic to moderate Covid-19 infection. The individual cases are shown in Table 2 (Tab. 2).

### Antibody levels

**Figure 1:**
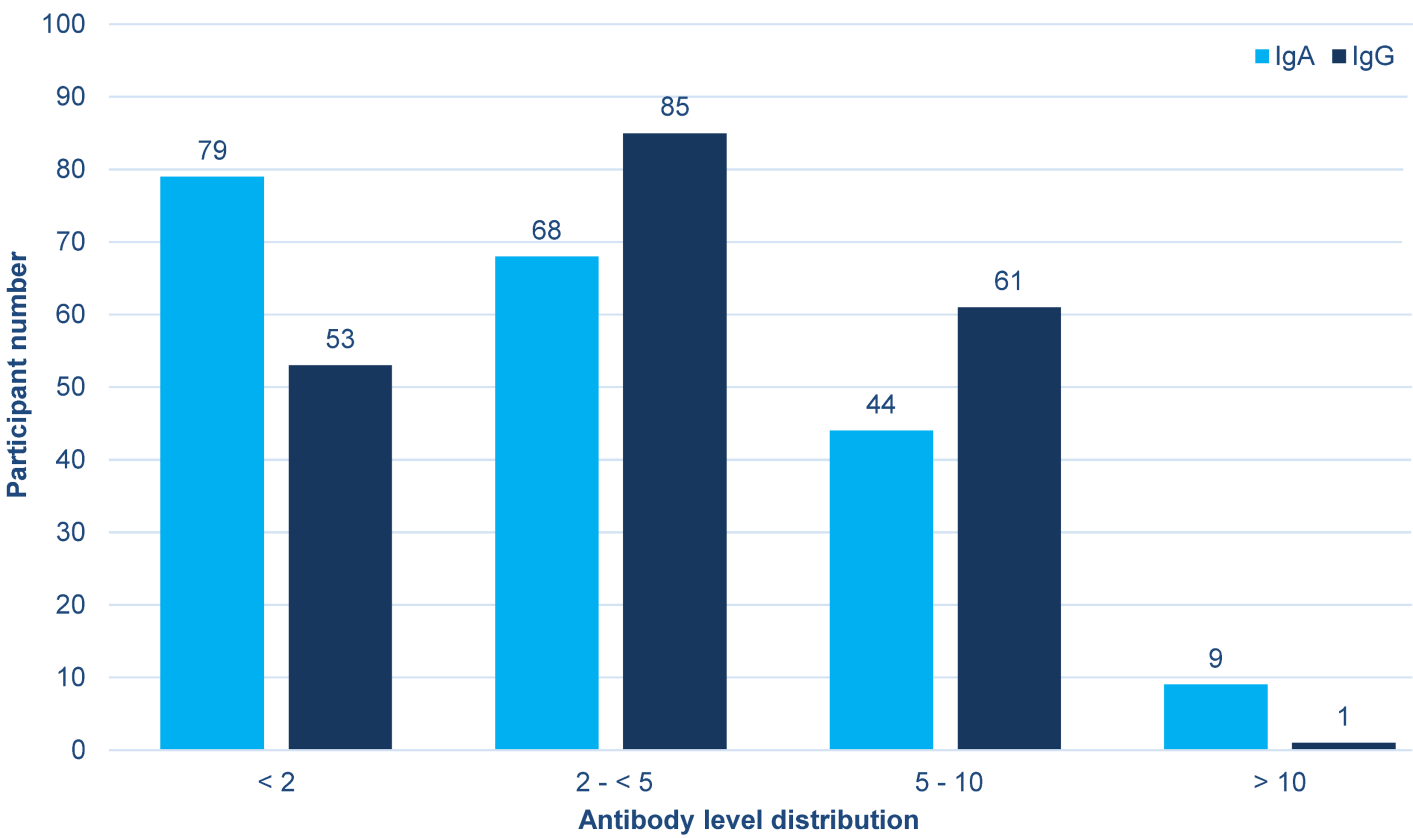
Antibody levels in the 200 study participants about three to four weeks after overcoming the disease (the participant numbers are given by antibody level groups on top of the columns)

The antibody levels are shown in Figure 1 (Fig. 1). The assay manufacturer defines values <0.8 as not measurable. Twenty-three persons were below this level for IgA (11.5%) and 16 persons for IgG (8%).

### Decision concerning vaccination

Before the results of antibody testing were available, 98 (49%) participants reported that they had already come to a decision concerning vaccination (Group 1), 88 (44%) participants reported that they had not yet come to a decision concerning vaccination (Group 2) and 14 (7%) participants did not answer (Group 3).

The results of the antibody tests were discussed personally with each study participant. The still-present attitude towards vaccination in the three groups was also queried and answered as follows:

- Group 1 (n=45 [45.92%]) against vaccination; n=39 [39.80%] for vaccination; indecisive in the sense of waiting/waiting for more recent scientific findings: n=14 [14.29%]. (The percentages are given for each group).
- Group 2 (n=45 [51.14%]) against vaccination; n=33 [37.50%] for vaccination; indecisive in the sense of waiting/waiting for more recent scientific findings: n=10 [11.36%]
- Group 3 (n=6 [42.86%]) against vaccination; n=3 [21.43%] for vaccination; indecisive in the sense of waiting/waiting for more recent scientific findings: n=5 [35.71%]

Initially, 44% of the employees (Group 2) were undecided about a vaccination. Decisions were made in this group at the second timepoint of the survey with knowledge of the antibody titers, but were only rarely influenced by the knowledge of the antibody titers (n=4; own statements). In all three groups, the decision to be vaccinated due to a change in the information base, e.g., antibody titers, but also due to several other factors, was made by six individuals, but mostly due to concerns about possible restrictions in social life or when traveling (n=9). Two people *de novo* decided against vaccination.

Of all 200 participants, 75 employees were finally in favor of a vaccination (37.5%), 96 were against vaccination (48%) and 29 could not make a definitive decision (14.5%).

### Sources of information

Information to facilitate vaccination decision-making on the corresponding homepages of the RKI or the STIKO, which literally contains the question: “Should people who have been through a SARS-CoV-2 infection or have recovered from COVID-19 be vaccinated?” (8) was used/known by 21 participants (8%).

### Occupation and impact on vaccination

In an attempt to analyze the position concerning vaccination, we separated the 200 persons into their occupational groups (Table 3 (Tab. 3)).

Even if there is a positive trend in terms of willingness to vaccinate, especially among physicians, and although the willingness to vaccinate seemed to be lowest among nurses, statistical testing was not possible due to the vastly different numbers of people in the various occupational groups.

### Age and impact on vaccination

In an attempt to evaluate their position on vaccination, we also separated the 200 persons into three cohorts according to their age (up to 25 yrs; 26–45 yrs, >45 yrs) (Table 4 (Tab. 4)).

There seemed to be a positive trend in terms of age: the older the participants were, the more willing they were to be vaccinated However, statistical testing is not possible due to the vastly different numbers of people in the various occupational groups.

## Discussion

Due to the high prevalence of COVID-19 infections in Thuringia during the “second wave”, it was not surprising that the number of infections was also high among the staff of our municipal clinic. Although the majority of participants reported “work” as the assumed most likely source of infection, this is an estimate which does not necessarily reflect reality, since household contacts are still regarded as the main source of transmission in the international literature [10]. As already reported for ICU staff [1], the willingness to be vaccinated is the highest among physicians and the lowest among nurses. Our results are comparable. However, to our knowledge, this is the first report about personnel in a municipal clinic with a past COVID-19 infection.

Our data also suggest a more positive attitude towards vaccination with increasing age. COVID-19 vaccination-rate monitoring in Germany (COVIMO Study) [11] by the RKI included 1006 adults as a representative random sample, of which 7.3% were medical personnel. In the first report [11] of the study, the authors also confirmed that the positive attitude towards vaccination is higher with higher age. In addition, the authors reported that neither gender nor occupation of the medical staff influenced the willingness to be vaccinated.

However, the term “medical staff” was generally used in that study, which was not further differentiated according to occupational groups. Although this was the case in our work, there does not necessarily have to be a contradiction. One problem with our study lies in the fact that the information regarding a vaccination decision was given by the participants. It cannot be clarified whether the vaccination actually took place. The study evaluation was also completed before vaccination with the Astra Zeneca vaccine were temporarily stopped (March 15^th^, 2021). 

Overall, the willingness to be vaccinated did not seem high in our study population. However, it should be remembered that these are people who have already had a COVID-19 infection. It would be interesting to compare it with a population of healthcare workers who have not yet been had this infection. If the willingness to be vaccinated were significantly higher in the latter group, a trust in the natural, acquired immunity in our population would most likely be the reason for the difference. It was already mentioned in the introduction that the duration and reliability of naturally acquired immunity has not yet been definitely clarified [6], [7]. The mutations that have occurred in the meantime also complicate the matter in this regard [12].

In terms of the antibody titers, we observed a relatively heterogeneous distribution of titer levels. Thirty-nine persons had IgA or IgG below the detectable range. This heterogeneous distribution has also been confirmed by other studies [13]. The severity of the disease (in the present study only asymptomatic to moderate cases) is evidently irrelevant for the level of the antibody titers. In fact, whether titer levels were detectable or not only induced very few affected persons to change their vaccination decision. 

In view of the previous individual case descriptions of cutaneous hyperesthesia in COVID-19 infection, we used the opportunity to specifically ask about these symptoms in a larger cohort. These symptoms were reported by 10 people, which allows the cautious conclusion of an incidence of 5% for a clinically asymptomatic to mild COVID-19 infection. The fact that only women were affected is more likely due to the high proportion of women in our study; cases in men are also described in the literature [14].

## Conclusions

In the case of medical personnel who have already suffered from a COVID-19 infection, the willingness to receive a vaccination tends to be highest among physicians and lowest in nurses, and increases with age. The antibody tests showed different titer levels and therefore did not correlate with the disease intensity in an asymptomatic to moderately-ill group. For the majority of those affected, knowledge of the antibody titer only reinforced the vaccination decision made beforehand, and thus did not contribute to a change in vaccination behavior. The specifically requested symptom of cutaneous hyperesthesia during COVID-19 infection is unexpectedly frequent at 5%.

## Notes

### Competing interests

The authors declare that they have no competing interests.

### Funding

There was no financial support.

### Acknowledgements

We are grateful to the persons who provided the blood samples to support our scientific research.

## Figures and Tables

**Table 1:**
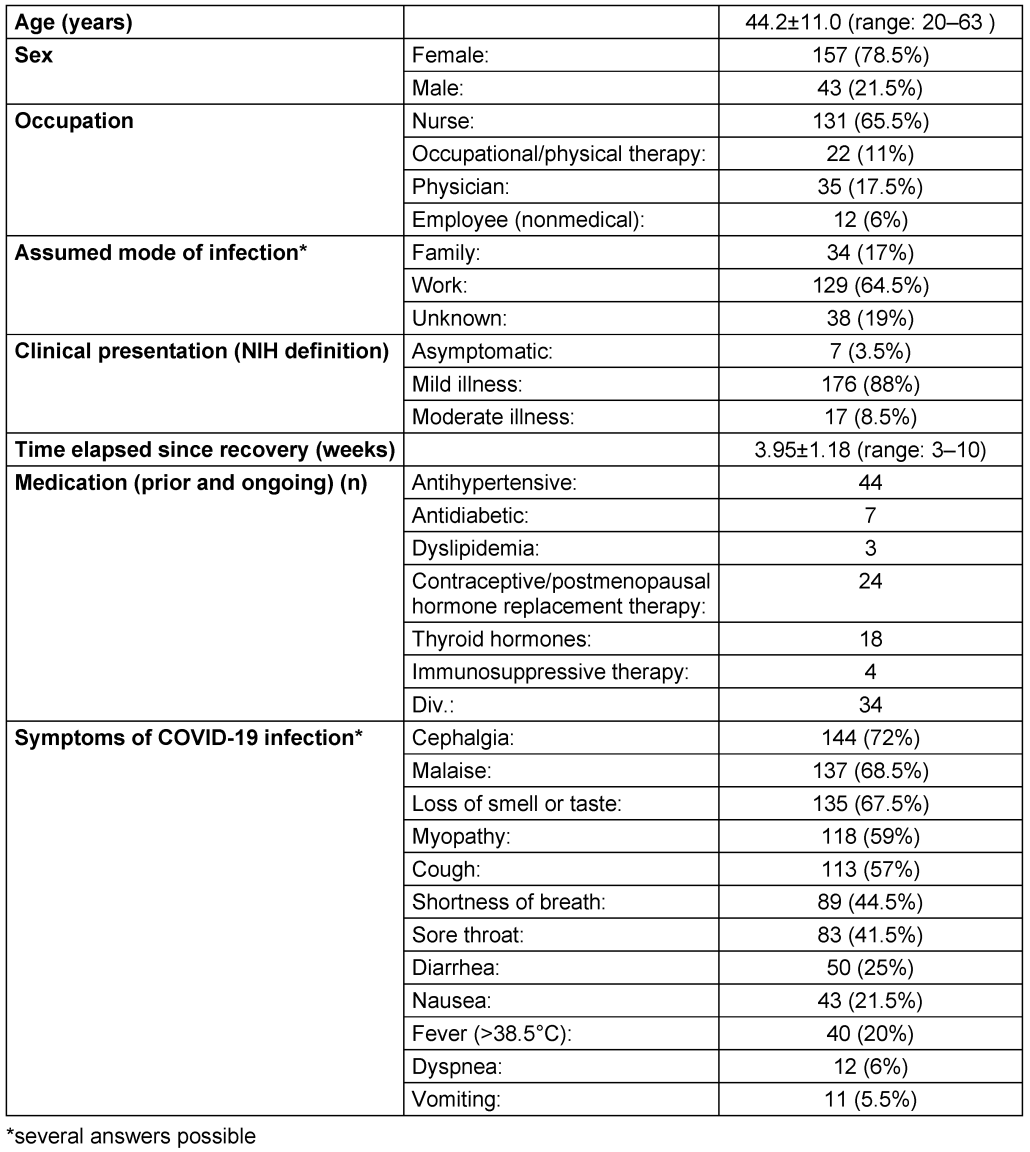
Characteristics of the 200 study participants

**Table 2:**
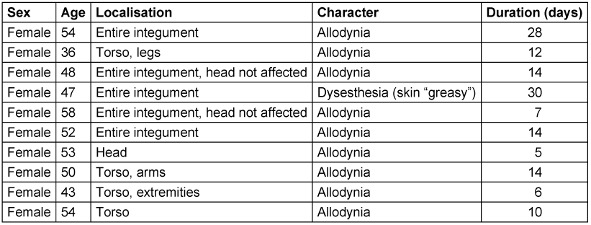
Detailed description of cutaneous hyperesthesia (localization, duration) in 10 of the 200 employees during the COVID-19 infection

**Table 3:**
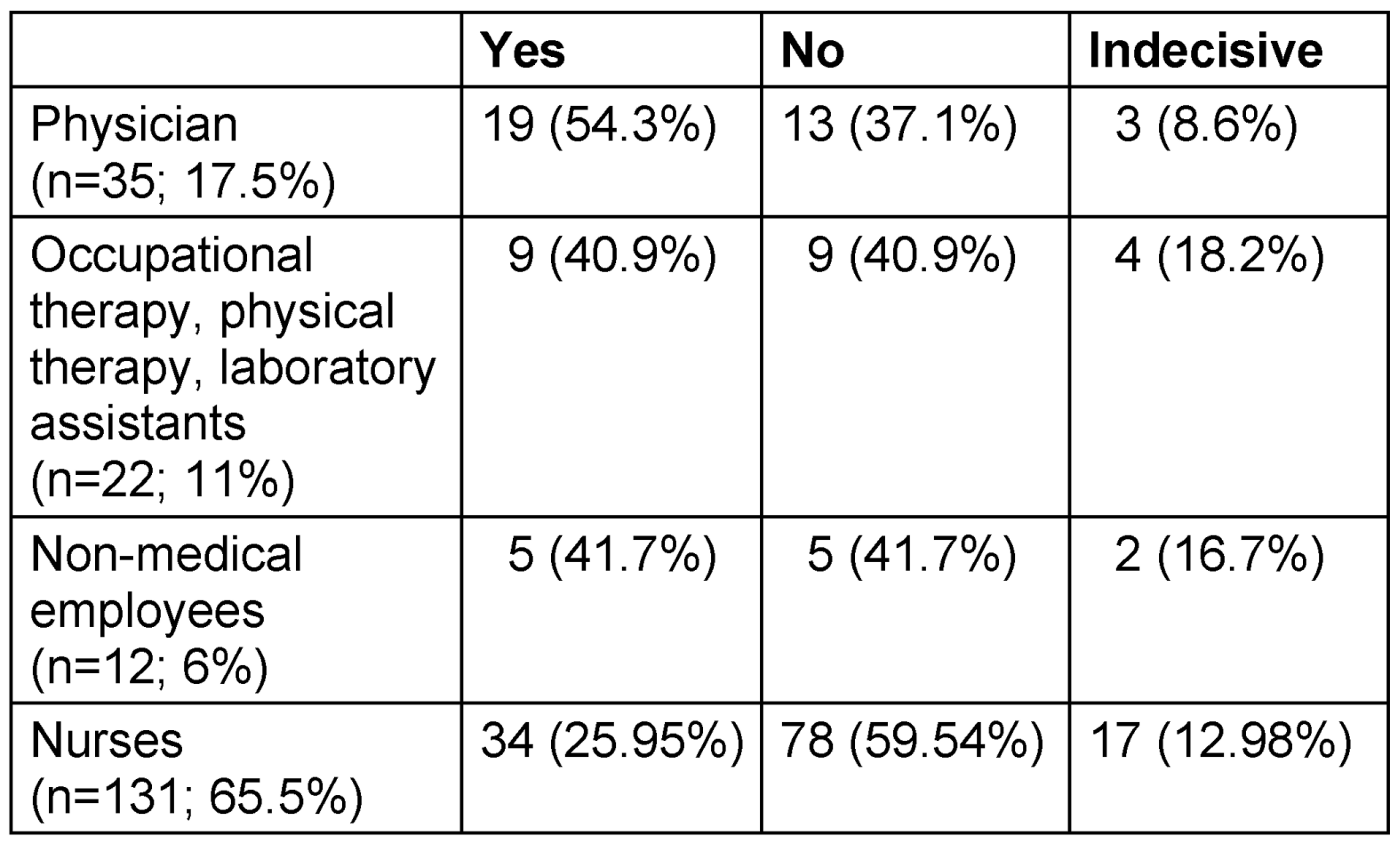
Occupational groups of the study participants

**Table 4:**
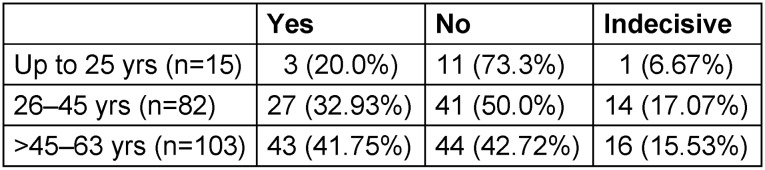
Allocation of study participants to age groups
